# The Establishment of an Efficient Callus Induction System for Lotus (*Nelumbo nucifera*)

**DOI:** 10.3390/plants9111436

**Published:** 2020-10-25

**Authors:** Xianbao Deng, Yaqian Xiong, Jing Li, Dong Yang, Juan Liu, Heng Sun, Heyun Song, Yunmeng Wang, Junyu Ma, Yanling Liu, Mei Yang

**Affiliations:** 1 Key Laboratory of Plant Germplasm Enhancement and Specialty Agriculture, Wuhan Botanical Garden, Chinese Academy of Sciences, Wuhan 430074, China; dengxianbao@wbgcas.cn (X.D.); xiongyaqian18@mails.ucas.ac.cn (Y.X.); yangdong@wbgcas.cn (D.Y.); liujuan@wbgcas.cn (J.L.); sunheng@wbgcas.cn (H.S.); songheyun17@mails.ucas.ac.cn (H.S.); 18838729704@163.com (Y.W.); majunyu181@mails.ucas.ac.cn (J.M.); liuyanling@wbgcas.cn (Y.L.); 2 Center of Economic Botany, Core Botanical Gardens, Chinese Academy of Sciences, Wuhan 430074, China; 3 College of Life Sciences, University of Chinese Academy of Sciences, 19A Yuquanlu, Beijing 100049, China; 4 School of Chemistry, Chemical Engineering and Life Sciences, Wuhan University of Technology, Wuhan 430070, China; jinli@whut.edu.cn

**Keywords:** lotus, callus, induction, factors

## Abstract

The lotus (*Nelumbo nucifera*) is one of the most popular aquatic plants in Asia, and has emerged as a novel model for studying flower and rhizome development, and primary and secondary metabolite accumulation. Here, we developed a highly efficient callus induction system for the lotus by optimizing a series of key factors that affect callus formation. The highest efficient callus production was induced on immature cotyledon and embryo explants grown on Murashige and Skoog (MS) basal medium containing an optimized combination of 3 mg/L 2,4-dichlorophenoxyacetic acid (2,4-D) and 0.5 mg/L 6-benzylaminopurine (6-BA). In addition, lotus callus induction was proven to be influenced by lotus genotypes, light conditions, the developmental stages of explants and the time of explant sampling. Collecting immature cotyledons from seeds of the genotype “Shilihe 1”, at 9 days post pollination, and to culture the explants in darkness, are proposed as the optimum conditions for lotus callus induction. Interestingly, highly efficient callus induction was also observed in explants of immature embryo derived aseptic seedlings; and a small amount of lotus benzylisoquinoline alkaloid (BIA) and obvious expression of BIA biosynthetic genes were detected in lotus callus.

## 1. Introduction

The Asian lotus (*Nelumbo nucifera*) is one of the only two extant species in the Nelumbonaceae family, cultivated throughout Asia for its high ornamental, edible and medicinal value [1]. Long term artificial selection and domestication has given rise to three categories of agriculturally useful lotuses, including the rhizome lotus, the flower lotus and the seed lotus [1,2]. The rhizome lotus develops enlarged edible rhizomes, and a few flowers bloom in summertime. The flower lotus is cultivated primarily for ornamental purposes; it blooms dense and attractive flowers and produces a few seeds. In contrast, the seed lotus develops numerous seed producing flowers, and is cultivated for harvesting fresh seedpods as fruits or mature seeds as food. In addition, the whole lotus plant is rich in bioactive benzylisoquinoline alkaloids (BIA) with significant medicinal properties, such as anti-obesity [3,4], anti-tumor [5,6], anti-diabetic [7] and anti-viral [8] activities.

Plant in vitro culturing is the widely implemented aseptic regeneration of cells, tissues, organs or whole plants under defined laboratory conditions for plant propagation, viral elimination, transgenic plant breeding and the preservation of rare plant genotypes or cells. In a plant tissue culture system, the basal medium supplies all the nutrients, energy and water necessary for explant growth, while the incubation systems provide desirable light and temperature conditions [9]. Through the addition of plant growth regulators, particularly the combinations of auxins and cytokinins, the growth and development of explants can be manipulated precisely. In addition, to develop a viable tissue culture system for certain plant species, their genotypes and explant types should also be carefully considered [10,11].

Due to its economic and medicinal value, the lotus has been extensively studied, particularly in the past decade [2,12,13,14,15,16,17,18,19]. With the release of its draft genome along with the finely improved version [20,21,22,23], the lotus is becoming an emerging horticultural model for studying flower color coloration, rhizome development, species phylogeny and primary and secondary metabolite biosynthesis [24]. However, the lotus is a severe recalcitrant plant for tissue culture as compared to other plant models, such as *Arabidopsis*, rice, tobacco, soybean and tomato plants, all of which have viable tissue culture and regeneration protocols developed [25,26,27,28,29]. This has severely limited functional genetic studies and production of transgenic lotus plants. Despite this, several studies on lotus tissue culture have been reported. Previous efforts were primarily done using rhizome apical tips, immature embryos and mature embryos as explants for plantlets micro-propagation [30,31,32,33]. Other attempts using apical buds and immature embryo tissues to induce callus and somatic embryogenesis have also been reported [34,35,36]. As a result, regenerated lotus shoots, plantlets and even transgenic plantlets were produced. These reported protocols were, however, hardly to be repeated. Lotus calluses developed based on these protocols easily got brown, and mostly died during the subculture and regeneration processes. Previous studies using bud tips, cotyledons and young leaves as explants reported moderate callus induction rates of approximately 20% within 12 weeks of culture [35,36]. Thus, both lotus callus induction efficiency and callus quality still need to be improved, to facilitate development of a viable lotus transformation and regeneration system.

In this study, we aimed to establish a reliable callus induction system for the lotus. The effects of key factors involved in lotus callus formation, such as lotus genotypes, explant sources, light and temperature conditions and the combination of plant growth regulators were assessed. BIA accumulation and the expressions of BIA biosynthetic genes were determined in lotus calluses. These results provide a useful starting point for the development of an integrated lotus transformation system based on somatic embryogenesis, and are valuable for building a cell culture system for in vitro production of medicinal BIAs.

## 2. Results

### 2.1. Callus Induction in Different Explants

Firstly, four different lotus tissues, including leaves, immature cotyledons, immature embryos and rhizome tips, were employed as explants for callus induction. All inductions were carried out on Murashige and Skoog (MS)medium [37] supplemented with 3mg/L 2,4-dichlorophenoxyacetic acid (2,4-D) and 1mg/L zeatin (ZT). After sterilization, approximately 50% of leaf explants and 20% of shoot tip explants remained sterile after 4 days of culture on MS medium, whereas over 95% of cotyledon and embryo explants were sterile.

**Figure 1:**
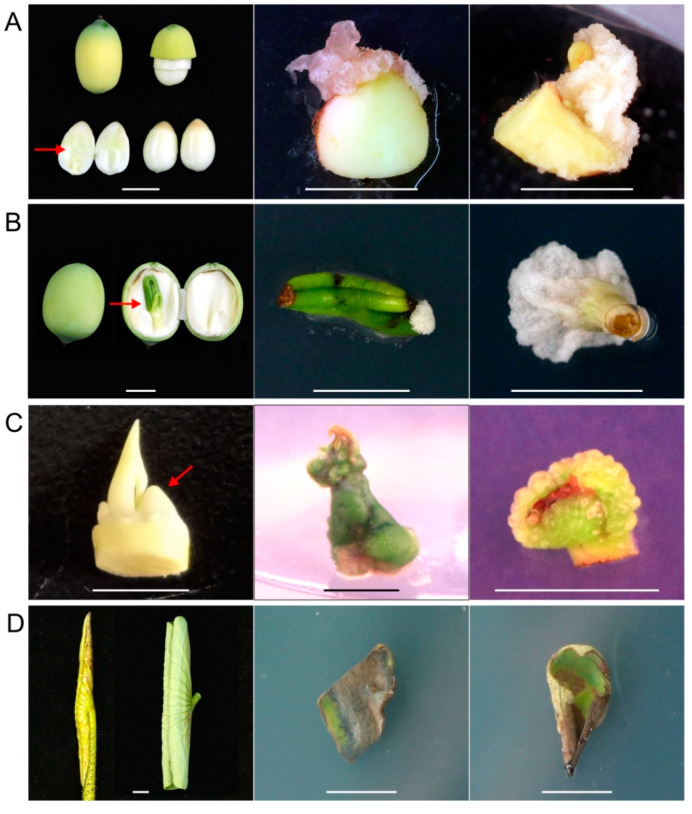
Callus induction in different lotus explants of the genotype “WBG_S1”. (**A**) Immature cotyledon explants from seeds at 9 days post pollination. (**B**) Immature embryo explants from seeds at 18 days post pollination. (**C**) Rhizome apical tip explants. (**D**) Leaf explants at the S3 and S4 developmental stages. Bars indicate the length of 1 cm. Red arrows indicate tissue/organ parts where explants were taken.

Among the four explants, immature cotyledons taken from seeds 9 days after pollination showed the earliest signs of callus formation at around 5 days post culture, followed by the sections of immature embryos taken from seeds 18 days after pollination, about 7 days after culture initiation. Both epicotyl and leaf sections from immature embryos were able to form calluses. After 3 weeks of culturing, callus induction was observed in approximately 20% of cotyledon explants (Figure 1A) and 12% of immature embryo sections, which were mostly induced from epicotyl and leaf sections (Figure 1B). These calli were primarily friable, soft and watery, with color varying from milky white to green depending on the light conditions applied (Figure 1A,B). Shoot tip cultures developed shoots at the top of the explants and calluses at the cut basal surfaces (Figure 1C). The calli developed from shoot tip explants were compact and green when cultured under light conditions, and were visible approximately 2 weeks after culture initiation. However, leaf explants did not develop any calluses until 4 weeks of culture (Figure 1D). Thus, immature cotyledons were the most ideal explants for callus induction. Moreover, immature cotyledons and embryos exhibited higher callus growth index and their sterilization was practically easier.

### 2.2. Callus Induction of Different Genotypes

**Figure 2:**
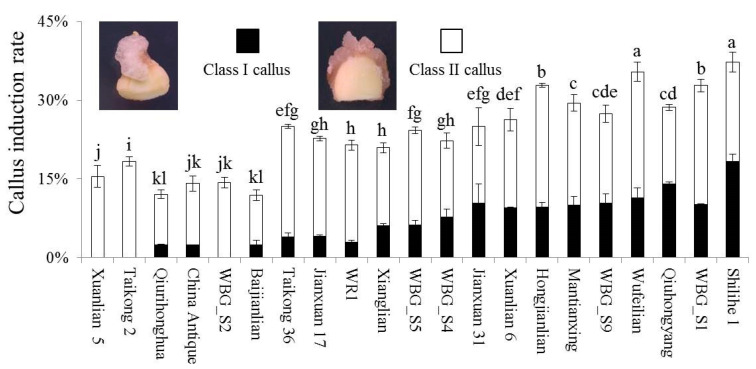
Effects of lotus genotypes on callus induction. Class I calluses indicate those of over 0.5 × 0.5 × 0.5 cm^3^, and class II indicates calluses smaller than 0.5 × 0.5 × 0.5 cm^3^. Different subcase letters indicate significant differences at *p* < 0.05.

As mentioned above, the best callus induction was achieved using immature lotus cotyledon. Thus, an immature cotyledon was used as the explant to investigate the effects of lotus genotypes on callus induction. Twenty-one lotus genotypes, including 6 flower lotus genotypes and 15 seed lotus genotypes, were employed for the study, and all culturing was done on MS basal medium containing 3 mg/L 2,4-D and 1 mg/L ZT. Great variation in the rate of callus induction was observed among these genotypes, which ranged from 11.96% in “Baijianlian” to 37.29% in “Shilihe 1”, with an average rate of 23.73%. In addition, “Shilihe 1” exhibited the highest induction rate of class I calluses, with a callus size of over 0.5×0.5×0.5 cm^3^, followed by “Qiuhongyang” and “Wufeilian” (Figure 2). Three genotypes, Xuanlian 5, Taikong 2 and WBG_S2, however, did not produce any class I calluses. Overall, lotus genotypes exhibited a great diversity in the rate of callus induction, with “Shilihe 1”, “Wufeilian” and “Qiuhongyang” being the most suitable genotypes for callus induction using immature cotyledons as explants.

### 2.3. Effects of Plant Growth Regulators on Callus Induction

In order to evaluate the effects of plant growth regulators on lotus callus induction, combinations of two auxins and two cytokinins were applied to MS basal medium (Table 1). A wide range of variation in the rate of callus induction was observed among the tested growth regulator combinations. Two combinations, 0.5 mg/L 2,4-D + 0.1 mg/L ZT and 1 mg/L 2,4-D + 0.1 mg/L ZT, did not induce any callus growth, whereas the other eight combinations promoted callus induction, with a total rate of callus induction ranging from 2.08% to 29.94%. Generally, at a uniform concentration, 2,4-D was a better auxin than 1-naphtalene acetic acid (NAA) for lotus callus induction, while when 3 or 7 mg/L 2,4-D was applied, 0.5 mg/L 6-benzylaminopurine (6-BA) was a better condition than 1 mg/L ZT. The best growth regulator combination was 3 mg/L 2,4-D + 0.5 mg/L 6-BA, which showed the highest induction rates of 14.37% and 15.56% in class I and class II calluses, respectively. This was followed by the combinations of 7 mg/L 2,4-D + 1 mg/L ZT and 3 mg/L 2,4-D + 1 mg/L ZT, which induced class I and II calluses at the rates of 10.09% and 9.28%, and 14.27% and 12.00%, respectively.

### 2.4. Optimizing Conditions for Lotus Callus Induction

**Figure 3:**
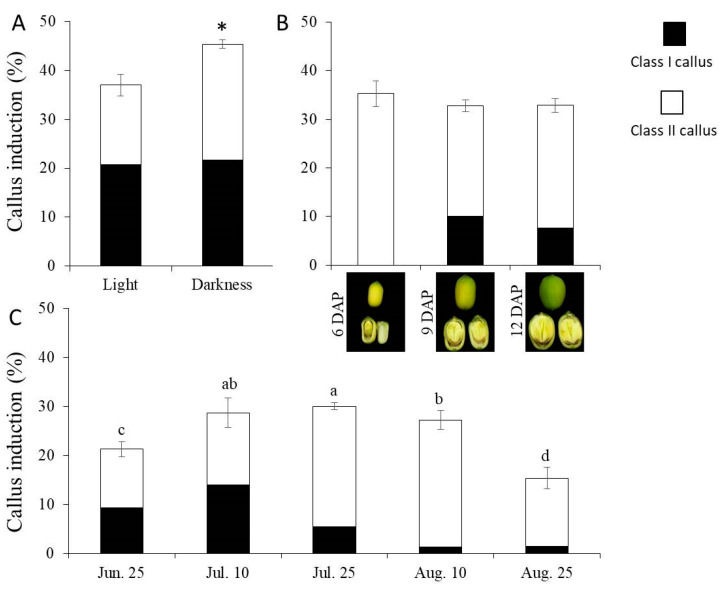
Evaluation of conditions affecting lotus callus induction in lotus genotype “WBG_S1”. The effects of light conditions (**A**), seed developmental stages for sampling immature cotyledon explants (**B**) and the sampling date (**C**) on lotus callus induction. Different lowercase letters and “*” indicate significant differences at *p* < 0.05.

Several conditions likely to affect callus induction in lotus were assessed. All experiments were carried out on MS medium supplied with 3 mg/L 2,4-D + 0.5 mg/L 6-BA. Initially, incubation in complete darkness and light conditions of 16 h light/8 h dark photoperiod was compared. The callus induction rate in complete darkness was 45.41%, which was significantly higher than 37.00% with the light conditions. However, the induction rate of class I calluses was comparable, with 20.73% and 21.71% in the light and darkness, respectively (Figure 3A). The effects of seed developmental stages on callus induction were also assessed. Cotyledons from lotus seeds at 6, 9 and 12 days after pollination (Figure 3B) were selected for callus induction. No significant difference in the total callus induction rate was observed in these stages. However, cotyledon explants from lotus seeds at 9 days after pollination showed the highest induction rate of 10.11% for class I callus, followed by lotus seeds at 12 days after pollination with the rate of 7.70%, whereas no induction of class I callus was observed on cotyledons from lotus seeds at 6 days after pollination (Figure 3B). Moreover, lotus seeds collected on different dates were tested for callus induction. As a result, lotus seeds collected in late July exhibited the highest callus induction rate, whereas those collected in early July recorded the highest rate of class I callus induction, followed by those collected in late June. In contrast, the lowest efficiency of class I callus induction was observed in cotyledon explants from seeds collected in August, which represent a later season of lotus seed production (Figure 3C). Taken together, collecting cotyledon explants from seeds 9 days after pollination in the early July and culturing in darkness presented the most ideal conditions for lotus callus induction.

### 2.5. Aseptic Seedlings as Explants for Callus Induction

**Figure 4:**
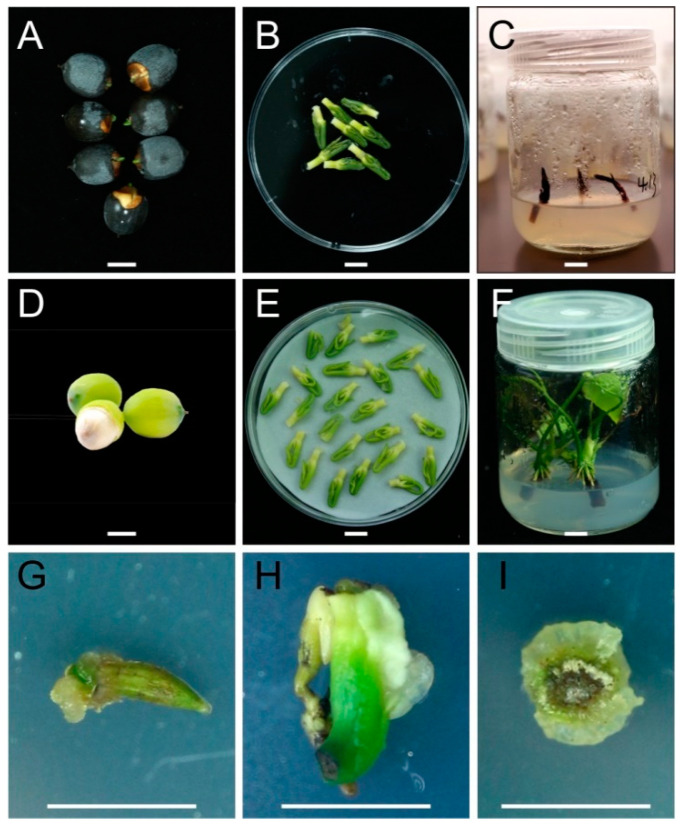
Regeneration of aseptic lotus seedlings for callus induction in lotus genotype “WBG_S1”. (**A**) Stored mature lotus seeds with part of episperm removed. (**B**) Embryos taken from mature lotus seeds. (**C**) An example of mature embryos that died during culture. (**D**) Immature seeds at 15 days after pollination. (**E**) Immature embryos taken from lotus seeds 15 days after pollination. (**F**) Aseptic seedlings developed from immature embryo cultures. (**G**–**I**) Callus induced from leaf sections, epicotyl-petile complex sections and epicotyl sections of aseptic seedlings respectively. Bars indicate the length of 1 cm.

As demonstrated above, complete sterilization of explants from lotus leaf and rhizome tips was less successful compared to immature embryos. Thus, embryos were cultured to produce aseptic lotus seedlings to be used for callus induction. Mature embryos were first taken from mature seeds for aseptic seedling cultivation (Figure 4A,B). After sterilization, contamination persisted in approximately 80% of mature embryos in the subsequent cultures. Successfully sterilized embryos, however, eventually died during subsequent culturing due to severe chemical injury of the apical meristem (Figure 3C). In contrast, over 95% of immature embryos taken from lotus seeds 18 days after pollination (Figure 3D,E) were free of contamination after sterilization, and grew well on MS medium supplied with 0.5 mg/L 6-BA + 0.5 mg/L NAA (Figure 3F).

These aseptic seedlings could directly be incised into sections for callus induction without the need for sterilization. Most sections, including those from leaves, petioles, epicotyls and epicotyl-petiole complexes, developed compact and green calluses (Figure 3G–I). Epicotyl explants exhibited the highest callus growth with an over 60% induction rate, followed by leaves and epicotyl-petiole complexes, with 35% and 30% rates of callus induction, respectively. Thus, aseptic lotus seedlings produced the most excellent explants for callus induction, whereas immature embryos collected from lotus seeds at 18 dap were considered the most ideal for culturing aseptic lotus seedlings.

### 2.6. Accumulation of Benzylisoquilonine Alkaloids (BIA) in Lotus Callus

**Figure 5:**
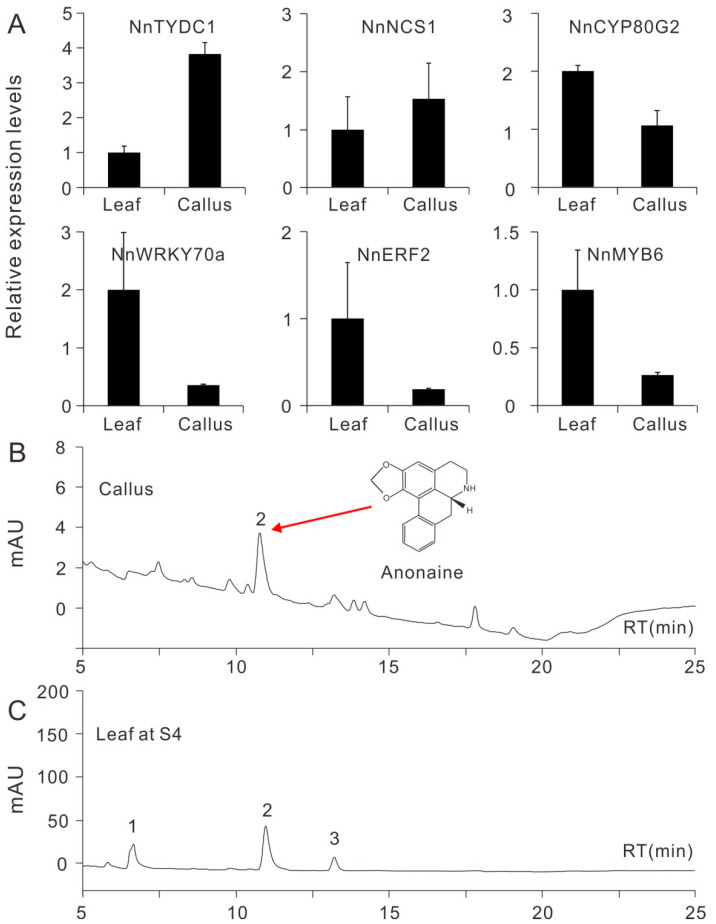
Relative expression of genes involved in benzylisoquilonine alkaloid (BIA) biosynthesis and the accumulation of BIA in lotus callus of the genotype “WBG_S1”. (**A**) Expression of three BIA biosynthetic genes and three transcription factors putatively involved in BIA biosynthesis. Data are means ± SE (*n* = 3). (**B**,**C**) HPLC measurements of BIA components and concentration in lotus callus and leaves at the developmental stage 4, respectively. Peaks 1–3 represent *N*-nornuciferine, anonaine and nuciferine respectively.

Plant tissue culture is a promising system for the production of highly valuable metabolites [38] in plants such as the lotus, which is known to be rich in bioactive BIAs [16]. To assess the potential application of lotus callus culturing in BIA production, we checked the expression of genes involved in BIA biosynthesis, and their contents in lotus calluses. Real-Time PCR results showed that three BIA biosynthetic genes, *NnTYDC1* (*tyrosine decarboxylase 1*), *NnNCS1* (*norcoclaurine synthase 1*) and *NnCYP80G2* [17], were obviously expressed in lotus calluses (Figure 5A), with comparable expression levels to those in lotus leaves at developmental stage 4 (S4) [16]. Moreover, *NnTYDC1* and *NnNCS1* exhibited relatively higher expression levels in the callus. Similarly, three transcription factors, *NnWRKY70a*, *NnERF2* and *NnMYB6*, potentially involved in BIA biosynthesis regulation [17,39], were also expressed in lotus calluses, but with relatively lower levels than in the leaves. HPLC results showed that lotus calluses accumulate low amounts of anonaine, with a concentration of 80.71 ug/g fresh weight (FW). In contrast, leaves of cv. “Hongjianlian” accumulated three BIA alkaloids, including *N*-nornuciferine, anonaine and nuciferine at S4, with a total BIA concentration of 2170 ug/g FW (Figure 5B,C).

## 3. Discussion

The lotus is one of the most important aquatic crops in China, with over 220,000 ha total cultivation area [1]. It is also an emerging horticultural model with a high-quality genome database available [20,21,22,23]. Although efforts have been made since the 1980s toward studying lotus tissue cultures [34], no feasible callus induction or regeneration protocols were available for this recalcitrant species. In this study, we established a reliable callus induction system by optimizing a series of key factors that influence lotus tissue culture.

Explant origin and plant genotypes were previously reported as key factors affecting callus induction [26,40,41]. Theoretically, any parts of a plant can be used as explants for callus induction; those taken from healthy plants or from plantlets and seedlings are the most suitable [42]. Among the four tested explants, young immature cotyledons were the most efficient in inducing lotus callus, showing evidence of early callus formation 5 days post culture, and an induction rate of 20% three weeks post culture. However, complete sterilization was less successful in lotus leaf and shoot tip explants, and both had relatively lower rates of callus induction as compared to immature cotyledon and embryo explants. In contrast, sections of immature lotus embryos were readily sterilized and displayed good callus induction, but with a relatively lower induction rate than immature cotyledons. Previously, lotus rhizome tips, cotyledons and young leaves have been used as explants for callus induction [35]. On MS medium containing 1.33 mg/L 2,4-D + 0.22 mg/L 6-BA, lotus rhizome tips exhibited higher callus induction rate (25% within 12 weeks of culture) than cotyledons and young leaves. However, the cotyledons used in their study were taken from mature lotus seeds, which in our analysis displayed low callus induction capacity (results not shown). Thus, lotus rhizome tips from the field are likely to be good explants for lotus callus induction if successful sterilization can be achieved.

Using immature cotyledons as explants, different lotus genotypes displayed great variation in callus induction, with the total rate of callus induction ranging from 11.96% to 37.29%. At 25 days post culture, seven lotus genotypes exhibited good callus induction capacity, with over 10% and 20% rates of class I callus induction and total callus induction, respectively. The three seed lotus genotypes, WBG_S2, Taikong 2 and Xuanlian5, however, did not produce any class I calluses in three weeks, and thus are unlikely to be suitable for callus induction.

Subsequently, the effects of auxin and cytokinin combinations on lotus callus induction were determined. A classical model developed on tobacco showed that a combination of high auxin and moderate cytokinin concentration promotes callus proliferation [43]. However, plant sensitivity to auixins or cytokinins varies among species. For example, synthetic 2,4-D auxin with concentrations ranging from 0.1 mg/L in cotton [44] to 40 mg/L in soybean plants [45] have been used to induce calluses in plants. Our results indicated that in the lotus, a low 2,4-D concentration of up to 1 mg/L could not induce callus formation, whereas a moderate concentration of 3 mg/L 2,4-D combined with 1 mg/L ZT or 0.5 mg/L 6-BA was the most efficient for callus induction. This is similar to the concentrations applied for callus induction in rice, *Arabidopsis* and maize [25,26,46]. Previous reports have shown that both the combinations of 1.33 mg/L 2,4-D + 0.22 mg/L 6-BA and 7.5 mg/L NAA + 0.11 mg/L TDZ induced a high quality callus in lotus [33,35,36]. Thus, the lotus seems adapted to a wide range of auxin concentrations in callus induction, whereas NAA and TDZ were superior for embryogenic callus production [36]. A high concentration of 2,4-D has been reported to inhibit callus cell division, and over-application of 2,4-D might cause herbicidal effects and slow down the callus induction process [47]. In addition, calluses inducted with high 2,4-D levels became watery and soft, which makes the subsequent regeneration process more difficult [48]. Thus, ideal amount of 2,4-D should be applied for lotus callus induction in order to guarantee both the callus quality and its volume.

Effects of other environmental factors such as light, which controls plant cell growth, development, morphogenesis and metabolism in tissue culture [49,50] were assessed. Light can affect callus development by adjusting endogenous auxin and gibberellin levels [51]. However, less effects of light on lotus callus induction were observed in our study. Immature cotyledon explants under darkness developed creamy and friable calluses, with a slightly higher rate of total callus induction than those grown in the light with a 45 µmol m^−2^ s^−1^, 16/8 h day/night photoperiod, which produced green and compact callus. Our results also suggest that immature cotyledon explants from lotus seeds collected 9 days after pollination in late June or early July are the most ideal for callus induction in the lotus.

Lotus explants collected from the natural environment are less likely to be successfully sterilized [35]. Thus, aseptic seedlings were regenerated from mature and immature embryo explants for callus induction. Sterilization of embryos taken from mature lotus seeds for aseptic lotus seedling regeneration was less successful in our study, but a recent report has shown that the mature embryos can be sterilized using a combination of ethanol, NaClO and PPM [33]. This could offer a great explant resource, because mature lotus seeds can be easily stored for many years, and are available year-around. In contrast, complete sterilization and successful aseptic seedling regeneration were achieved for embryos collected from immature lotus seeds 18 days after pollination. Interestingly, high quality calluses were developed from aseptic seedling sections, with an induction rate over 60%, thereby providing a better alternative for lotus callus induction.

It is worth noting that anonaine, one of the medicinally valuable BIAs identified in lotus leaves, was detected in lotus calluses, although its concentration was relatively low in comparison to that in leaves. A consistent expression of BIA biosynthetic genes was also detected in the lotus callus, suggesting that with optimized conditions and appropriate elicitors, the lotus callus induction system developed in this study could potentially be used to produce BIAs in vitro.

In conclusion, this study has highlighted a series of key factors that affect lotus callus induction, including plant genotypes, explant sources, auxin/cytokinin ratios, light, the developmental stages of explants and time of explant sampling. Under the optimized conditions, highly efficient callus induction was achieved from immature cotyledons or aseptic seedling explants. These results will be valuable for designing future transgenic and regeneration systems in the lotus based on somatic embryogenesis.

## 4. Material and Methods

### 4.1. Plant Materials

A total of 21 Asian Lotus genotypes were used in this study, including “Xuanlian 5′, “Xuanlian 6′, “Taikong 2′, “Taikong 36′, “Jianxuan 17′, “Jianxuan 31′, “WBG_S1′, “WBG_S2′, “WBG_S4′, “WBG_S5′, “Baijianlian”, “Hongjianlian”, “Qiurihonghua”, “Qiuhongyang”, “China Antique”, “WBG_S9′, “Xianglian”, “WR1′, “Wufeilian”, “Shilihe 1” and “Mantianxing”. All genotypes were cultivated separately in concrete pond in the lotus germplasm resource center of Wuhan Botanical Garden, Chinese Academy of Sciences (30°32′ E–116°25′ N). All genotypes were used for testing the effect of lotus genotype on callus induction, whereas “WBG_S1′ was used for all other experiments.

### 4.2. Preparation of Lotus Explants

Four types of lotus explants taken from the “WBG_S2” genotype were tested for callus induction, including: (1) leaves at developmental stage S3 or S4 [16]; (2) rhizome tips; (3) immature cotyledons taken from seeds 9 days after pollination; and (4) immature embryos taken from seeds 18 days after pollination. All explants were used to test the effects of explant types on lotus callus induction, whereas only explant 3 was used for testing the effects of lotus genotypes, growth regulator combinations and other conditions on lotus callus induction. All explants were first washed thoroughly with tap water, submerged in 70% ethanol for 30 s and then surface sterilized in 0.1% (*w*/*v*) mercuric chloride (HgCl_2_) for 8 min, followed by a 5-time final rinse with sterile Mill-Q water. For leaf explants, sections of size approximately 0.5 × 0.5 cm^2^ were prepared, and for rhizome tip explants, shoot tips about 0.5 cm in length were used. To obtain immature cotyledon and embryo explants, both outer and inner seed sheaths were carefully removed. Immature cotyledons were sliced into two halves, and embryos were divided into epicotyl sections, petiole sections, leaf sections and petiole-epicotyl complexes before planting in solid medium for callus induction.

### 4.3. Conditions for Callus Induction

Lotus explants were cultivated on MS basal medium supplied with various combinations of auxins [2,4-D (Mackin, Shanghai, China), NAA (Biosharp, Guangzhou, China)] and cytokinins [6-BA (Biosharp, Guangzhou, China), Zeatin (Biosharp, Guangzhou, China)]. All media contained 30g/L sucrose (Biofroxx, Guangzhou, China) and 8 g/L agar (Biofroxx, Guangzhou, China), with the pH set at 5.8 before autoclaving (121 °C for 20 min). Cultures were set in the growth room at 25 °C under a 16/8 h day/night photoperiod or complete darkness. Light intensity was adjusted to approximately 45 µmol m^−2^ s^−1^ with led lights (PHILIPS, Guangzhou, China).

### 4.4. Quantification of Lotus Alkaloids and Quantitative RT-PCR

Total alkaloids in lotus calluses were extracted and quantified according to our previously reported protocol [16]. In brief, 600 mg fresh leaf or callus samples were ground into powder in liquid nitrogen, and extracted with 7 mL of extraction buffer (0.3M HCl:methalnol, 1:1, *v*/*v*) twice, under a 30 min sonication. After centrifugation at 10,000 g for 10 min, two supernatants were combined and diluted to a final volume of 15 mL with extraction buffer. Alkaloid extracts were filtered with 0.22 μm membranes (Shanghai New Asia Purification Device Factory, Shanghai, China) and subjected to high-performance liquid chromatography (HPLC) for concentration quantification.

RNA extraction was carried out with the RNAprep Pure Plant Kit (Tiangen Biotec, Beijing, China) according to the instructions of the manufacturer. cDNA synthesis was conducted using cDNA Synthesis SuperMix (TransGen Biotech, Beijing, China) by following the manufacturer’s instructions. Expressions of BIA biosynthetic genes were quantified using StepOnePlus^TM^ Real-Time PCR System (Applied Biosystems) and a 2 × SYBR Green II Master Mix (Takara, Dalian, China). The relative expression of genes was calculated using the 2^−ΔΔct^ method, and the expression of a lotus *Actin* gene was used as an internal control. All experiments were conducted in three biological replicates, and primers used in this study are listed in Table S1.

### 4.5. Data Analysis

For each experiment, at least 100 explants per genotypes or explant type were tested, and all experiments were carried out in triplicate. Induction of lotus callus was recorded 20–40 days post culture. Lotus calluses were classified into two types based on size: class I, calluses larger than 0.5 × 0.5 × 0.5 cm^3^, and class II, calluses smaller than 0.5 × 0.5 × 0.5 cm^3^ (Figure 1). The callus induction rate was defined as: callus induction rate (%) = (number of explants produced callus/total number of explants cultured) × 100%. Statistical significance was calculated using SPSS software (Version 22.0) with one-way analysis of variance (ANOVA) method, and significant difference was defined by Duncan’s multiple range test at *p* < 0.05.

## Figures and Tables

**Table 1:** Effects of plant growth regulators on class I, class II and total rate of callus induction in lotus cotyledons explants.

Plant Growth Regulators (mg/L)				Class I Callus (%)	Class II Callus (%)	Total Callus (%)
NAA	6-BA	2,4-D	ZT			
1	0.5	–	–	2.08 ± 1.80 ^e^	0.00 ± 1.10 ^f^	2.08 ± 1.80 ^f^
3	0.5	–	–	9.41 ± 1.10 ^b^	4.65 ± 1.33 ^e^	14.06 ± 1.65 ^d,e^
5	0.5	–	–	4.49 ± 0.28 ^d^	8.98 ± 0.56 ^d^	13.47 ± 0.84 ^e^
7	0.5	–	–	6.89 ± 0.47 ^c^	9.19 ± 0.63 ^c,d^	16.08 ± 1.10 ^d^
–	0.5	7	–	9.20 ± 0.44 ^b^	10.73 ± 0.52 ^b,c^	19.93 ± 0.96 ^c^
–	0.5	3	–	14.37 ± 0.15 ^a^	15.56 ± 0.88 ^a^	29.94 ± 0.73 ^a^
–	–	3	1	9.28 ± 0.81 ^b^	12 ± 1.18 ^b^	21.28 ± 1.46 ^c^
–	–	7	1	10.09 ± 0.15 ^b^	14.27 ± 1.26 ^a^	24.36 ± 1.11 ^b^
–	–	0.5	0.1	0.00 ± 0.00 ^g^	0.00 ± 0.00 ^g^	0.00 ± 0.00 ^g^
–	–	1	0.1	0.00 ± 0.00 ^g^	0.00 ± 0.00 ^g^	0.00 ± 0.00 ^g^

Note: Data were collected in lotus genotype “WBG_S1” 20 days after culture. Each value represents mean ± SE (*n* = 3), and the different superscripted letters indicate significance level at *p* < 0.05. NAA: 1-naphtalene acetic acid; BAP: 6-benzylaminopurine; 2,4-D: 2,4-dichlorophenoxyacetic acid; ZT: Zeatin.

## Supplementary Materials

The following are available online at https://www.mdpi.com/2223-7747/9/11/1436/s1, Table S1: Primers used for quantitative RT-PCR.
