# Supplementary material for: The Establishment of an Efficient Callus Induction System for Lotus (Nelumbo nucifera)
Source: Plants (Basel). 2020 Oct 25;9(11):1436. doi: 10.3390/plants9111436 (PMC7693671; doi:10.3390/plants9111436)
Supplement: Supplementary file 1 [file plants-09-01436-s001.pdf]

Table S1. Primers used for quantitative RT-PCR.

No	Name	Sequence (5′-3′)
1	NnACTIN_F	CTCCGTGTTGCCCCTGAAG
2	NnACTIN_R	CCAGCAAGGTCCAACCGAAG
3	NnTYDC1_F	GCAAGACGTGCAGAAAGAAATTATCC
4	NnTYDC1_R	GCAGCAGGCGAAGACATCCAA
5	NnNCS1_F	GAGCTGCCCAGACTCTTCGTC
6	NnNCS1_R	TACCTTCTCCCGGTGTTGATG
7	NnCYP80G2_F	AGCGTCCGAATTAAGGGCTAT
8	NnCYP80G2_R	ATCAGTTCCGATACTTTCTCCTCT
9	NnWRKY70a_F	CAGGAATGCAAAGAGGTGGAGA
10	NnWRKY70a_R	ACCTGAGTACACGCCCGAGAT
11	NnERF2_F	TGTTCAGGCGAGGAATCAAGG
12	NnERF2_R	ACATTACAAGCGTCACGGAGCAT
13	NnMYB6_F	CAGCAGCAACAGCAATACTAACAA
14	NnMYB6_R	CAGGATGACTTCTCCCACGAT
